# Second-order grey-scale texture analysis of pleural ultrasound images to differentiate acute respiratory distress syndrome and cardiogenic pulmonary edema

**DOI:** 10.1007/s10877-020-00629-1

**Published:** 2020-12-12

**Authors:** Claudia Brusasco, Gregorio Santori, Guido Tavazzi, Gabriele Via, Chiara Robba, Luna Gargani, Francesco Mojoli, Silvia Mongodi, Elisa Bruzzo, Rosella Trò, Patrizia Boccacci, Alessandro Isirdi, Francesco Forfori, Francesco Corradi, UCARE (Ultrasound in Critical care and Anesthesia Research Group)

**Affiliations:** 1 Anaesthesia and Intensive Care Unit, E.O. Ospedali Galliera, Genoa, Italy; 2 Department of Surgical Sciences and Integrated Diagnostics, University of Genoa, Genoa, Italy; 3 Department of Clinical, Surgical, Diagnostic and Pediatric Sciences, Intensive Care Unit, Fondazione Policlinico San Matteo IRCCS, University of Pavia, Pavia, Italy; 4 Department of Cardiac Anesthesia and Intensive Care Fondazione Cardiocentro Ticino, Lugano, Switzerland; 5 Anesthesia and Intensive Care, Policlinico San Martino, IRCCS for Oncology and Neuroscience, Genoa, Italy; 6 Institute of Clinical Physiology, National Research Council, Pisa, Italy; 7 Anaesthesia and Intensive Care, San Matteo Hospital, Pavia, Italy; 8 Department of Informatics, Bioengineering, Robotics and System Engineering (DIBRIS), University of Genova, Genoa, Italy; 9 Department of Surgical, Medical, Molecular Pathology and Critical Care Medicine, University of Pisa, Pisa, Italy

**Keywords:** Artificial intelligence, Computer aided diagnosis, Quantitative lung ultrasonography, Lung ultrasonography, Heart failure, Acute respiratory failure

## Abstract

Discriminating acute respiratory distress syndrome (ARDS) from acute cardiogenic pulmonary edema (CPE) may be challenging in critically ill patients. Aim of this study was to investigate if gray-level co-occurrence matrix (GLCM) analysis of lung ultrasound (LUS) images can differentiate ARDS from CPE. The study population consisted of critically ill patients admitted to intensive care unit (ICU) with acute respiratory failure and submitted to LUS and extravascular lung water monitoring, and of a healthy control group (HCG). A digital analysis of pleural line and subpleural space, based on the GLCM with second order statistical texture analysis, was tested. We prospectively evaluated 47 subjects: 16 with a clinical diagnosis of CPE, 8 of ARDS, and 23 healthy subjects. By comparing ARDS and CPE patients’ subgroups with HCG, the one-way ANOVA models found a statistical significance in 9 out of 11 GLCM textural features. *Post*-*hoc* pairwise comparisons found statistical significance within each matrix feature for ARDS *vs*. CPE and CPE *vs*. HCG (*P* ≤ 0.001 for all). For ARDS *vs*. HCG a statistical significance occurred only in two matrix features (correlation: *P *= 0.005; homogeneity: *P* = 0.048). The quantitative method proposed has shown high diagnostic accuracy in differentiating normal lung from ARDS or CPE, and good diagnostic accuracy in differentiating CPE and ARDS. Gray-level co-occurrence matrix analysis of LUS images has the potential to aid pulmonary edemas differential diagnosis.

**Supplementary Information:**

The online version of this article (10.1007/s10877-020-00629-1) contains supplementary material, which is available to authorized users.

## Introduction

Acute hypoxemic respiratory failure secondary to pulmonary edema is a life-threatening condition frequently found in in intensive care units [1]. Pulmonary edema is an abnormal accumulation of extravascular lung water (EVLW), which may occur when capillary permeability or hydrostatic pressure are increased. The former is the mechanism underlying non-cardiogenic pulmonary edema as in adult respiratory distress syndrome (ARDS), whereas the rise in hydrostatic pressure represent the underlying cause of dyspnea in patients with heart failure and cardiogenic pulmonary edema (CPE) [2, 3].

Discriminating ARDS from CPE may be challenging in critically ill patients [4, 5], as there could be both overlapping clinical signs and confounders, including past history of respiratory or cardiac diseases. Echocardiography is a powerful tool in the discrimination between CPE and ARDS [4], but requires estimation of the left ventricular (LV) diastolic function and left atrial pressure. However, echocardiography carries some limitations: (1) absolute values are not meaningful, especially in presence of chronic heart failure, but it would rather require a monitoring of filling pressures; (2) may not always be feasible in the critically ill patients (due to potential windows quality limitation); (3) and may be out of reach for clinicians not trained in comprehensive echocardiography.

Lung ultrasonography (LUS) is nowadays widely adopted to assess lung aeration and extravascular water content [6, 7]. One study suggested that lung ultrasonography (LUS) may help differentiate between cardiogenic and non-cardiogenic pulmonary edema [8], although the results were not confirmed in other studies [9–11]. LUS semiotics of interstitial diseases is mainly based on presence, number and distribution of artifacts generated at the level of the pleural line, namely B-lines, reflecting the loss of lung aeration regardless the etiology, on which all the scoring systems are based [12, 13]. The main difference between the LUS pattern of CPE and ARDS reflects the pathophysiology: CPE is characterized by a homogenous distribution of interstitial syndrome (therefore of B-lines) whereas ARDS presents interstitial syndrome/loss of aeration (B-lines) with spared area (normal LUS pattern) and sub-pleural or lobar consolidations. The scoring systems validate so far have been semi-quantitative [14, 15].

Starting from the assumption that pleural and subpleural findings represent the main difference between ARDS and CPE [8, 13, 16] we developed a new algorithm for the specific analysis of the pleural line and the immediate subpleural space, based on the gray-level co-occurrence matrix (GLCM) and with a second order statistical method of texture analysis. A well-established analysis methodology has already been studied with prostate, breast, and endometrial ultrasound images [17–19]. To our knowledge, this has not been applied yet to LUS images obtained from patients with acute respiratory failure. The aim of this study was to investigate different features of gray-level co-occurrence matrix in order to assess their diagnostic accuracy in the differentiation of a series of LUS images form ARDS or CPE patients.

## Patients and methods

### Subjects

We prospectively recruited a sample of twenty-four critically ill patients admitted to the intensive care unit due to cardiogenic shock related to myocardial infarction or septic shock with acute respiratory failure with and clinical indication to EVLW monitoring with the trans-pulmonary thermo-dilution technique. LUS was used for clinical monitoring according to the standard clinical practice. ARDS complying with Berlin definition [4], was diagnosed in patients with septic shock by EVLWi > 10 mL/kg and pulmonary vascular permeability index (PVPI) ≥ 3.0 [20]. Patients with cardiogenic shock, EVLWi > 10 mL/kg, PVPI < 3.0 and echocardiographic signs of increased left atrial pressure, inferred by E/A < 0.75 or > 0.75 or E/A > 1.5 associated with E/E′ > 10, were diagnosed as CPE [21]. All patients were sedated with continuous propofol infusion and mechanically ventilated with a tidal volume of 6 mL/kg of predicted body weight, and positive end expiratory pressure of 5 cmH_2_O at the time of image acquisition. Twenty-three healthy subjects were used as controls. The local ethical committee approved the study (Ethics Committee for Liguria Region n. 041/2018).

### LUS

Images and videoclips were acquired with Esaote MyLab alpha or Mindray DC-N3 ultrasound machines, using a high-frequency (10 MHz) linear probe, with the patient in the supine position. Transversal scans (parallel to the ribs) were adopted in order to visualize the pleural line without any rib shadowing [22]. The focus was set at the level of the pleural line, and 2nd harmonic removed to avoid artifacts attenuation. The probe was placed perpendicular to the scanning surface with minimal pressure applied to the footprint. All B-mode images were saved in 8-bit grey scale DICOM format and the intensity ranged from 0 to 255. Six standard areas of each hemi-thorax were identified relative to sternum and axillary lines: anterior, lateral, and posterior, each one divided into upper and lower quadrants. The most pathological scan area of each single quadrant was considered representative of the whole quadrant itself, and acquired as a video clip. A progression from A pattern (normal) to limited B-lines (involving ≤ 50% of the pleural line) to predominant B-lines (> 50% of the pleural line) to consolidation was the reference for severity in abnormality that guided this choice [22].

Second-order grey-scale texture analysis was performed with a dedicated software by technicians (blind to the clinical diagnosis), on a still image, selected from each video clip as most representative of the corresponding dynamic LUS pattern. The mean of the findings of the 12 areas was retained for subsequent statistical analysis.

### Automated scoring algorithm and grey-scale texture analysis

**Fig. 1:**
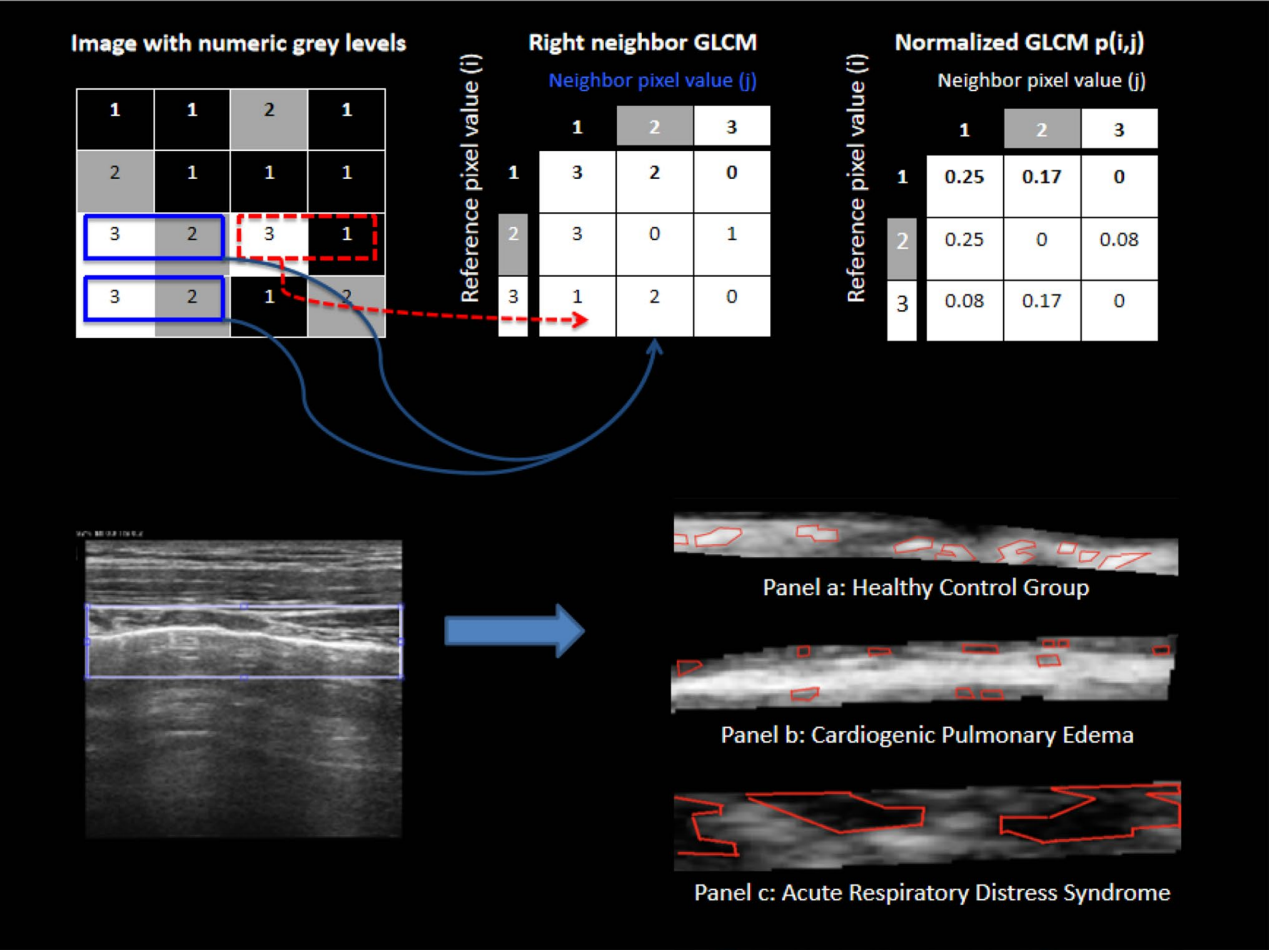
In second-order statistical texture analysis, information on texture is based on the probability of finding a pair of grey-levels at random distances and orientations over an entire image. This is done through computing Grey-Level Co-Occurrence Matrices (GLCMs). The entries in a GLCM are the probability of finding a pixel with grey-level *I*, having set a distance *d* and angle *θ* from a pixel with a grey-level *j*, that is: *P*(*i, j*:*d, θ*). An essential component of this framework is pixel connectivity, where each pixel has eight nearest-neighbours connected to it, except at the periphery. As a result four GLCMs are required to describe the texture content in the horizontal (*PH *= 0°), vertical (*PV *= 90°) right (*PRD *= 45°) and left-diagonal (*PLD *= 135°) directions. The information extracted from these matrices can be used for computing textural features, specifically designed for this purpose which are sensitive to specific elements of texture. Panel a: In the image, a local zoom of a healthy pleural line area highlights that brighter (white) regions are present against a “darker” (light grey) background that results in high positive “Cluster Shade” values. Panel b: shows a local zoom in the pleural line area of an acute cardiogenic pulmonary edema subject (globally looking similar to a healthy one to the human eye) presents darker (light/dark grey) regions against a lighter background. This results in negative “Cluster Shade” values. Moreover, a local zoom of the pleural line area shows small regions with uniform dark grey intensity resulting in low “Correlation”. Panel c: in this image, local zoom of an ARDS pleural line area shows large regions with uniform dark grey intensity resulting in high “Correlation”

We used texture analysis with second-order statistics because it provides unique information on the structure of the texture in the image being investigated. The analysis is made on clips in DICOM format, and consists of computing grey-level co-occurrence matrices with entries being the probability of finding a pixel with grey-level “*i*” at set distance “*d*” and angle “*θ*” from a pixel with a grey-level “*j*”, P(i, j:d, θ). An essential component of this framework is pixel connectivity: each pixel has eight nearest-neighbours connected to it, except at the periphery. As a result, four grey-level co-occurrence matrices are required to describe the texture content in the horizontal (PH = 0°), vertical (PV = 90°), right (PRD = 45°) and left diagonal (PLD = 135°) directions (Fig. 1). Grey-level co-occurrence matrices were computed averaging along all four directions, thus obtaining a direction-invariant, symmetrical matrix. The information extracted from these matrices were used for computing the features that are sensitive to specific elements of texture. The grey-level co-occurrence matrices and texture features computed in this way were not reported cause significant errors due to redundancy. These features are described in the Table 1, including three additional sum parameters.

**Table 1:** Computed features that were sensitive to specific elements of the texture content

Computed Feature	Description
Contrast	A measure of the local variations in an image
Shade	A measure of the skewness of the grey-level co-occurrence matrix giving large positive values when “lighter” areas are present on a “darker” background, and large negative values when “darker” areas are present on a “lighter” background
Entropy	A measure of information content. It measures the randomness of intensity distribution. A homogeneous scene has a high entropy
Variance	The grey level variability of the pixel pairs and is a measurement of heterogeneity
Mean	A measure of the mean grey intensity of the image, calculated for the columns and rows of the matrix
Correlation	A measure of grey level linear dependence between the pixels at the specified positions relative to each other
Energy	A measure of global homogeneity of an image, also known as angular second moment
Homogeneity	A measure of local homogeneity of an image, also known as inverse difference moment
Mean sum	A measure of the mean of the grey level sum distribution of the image
Entropy sum	A measure of disorder related to the grey level sum distribution of the image
Variance sum	A measure of the dispersion of the histogram obtained by considering the sum of near grey levels. This feature goes beyond the human visual interpretation

### Parameter setup

Starting from the analysis of a region of interest surrounding and including the pleural line, we tested various sets of parameters for the grey-level co-occurrence matrix computation, namely, number of grey levels (*Ng*), distance between pixel pairs (*d*), and direction (*θ*). For *Ng*, we found that 16 provides a good balance between computation time and preservation of image information and values up to 64 did not provide significant differences in outcome. For displacement vector *d*, we found that values from 1 to 4 permitted to highlight significant variations in detail. For direction, we used the whole set of angles (0°, 45°, 90°, 135°), because orientation could produce either similar or distinctively different grey-level co-occurrence matrix, depending on textures.

### Software development and analysis of clinical cases

For of the analysis of patients’ images, where the exact position of pleural line is not known in advance, we applied an interactive selection of a rectangular region of interest around the line. Furthermore, to delineate the pleural region more precisely, we allowed the user select a polygonal region of interest surrounding the line and following its course with exclusion of rib images, if any. For each frame in a region of interest, we computed four gray-level co-occurrence matrices and the related Haralick’s textural features. These were the following: contrast, variance, cluster prominence, cluster shade, entropy, correlation, homogeneity, energy, column means and standard deviations, row means and standard deviation, sum average, sum entropy, sum variance. Since there was no significant inter-distance or inter-direction variability among the values computed from each gray-level co-occurrence matrix we averaged all values of each feature to obtain a single value per frame.

### Thermo-dilution method

A VolumeView™ catheter (Edwards Lifesciences) for trans-pulmonary thermo-dilution measurements was inserted into the left/right femoral artery and connected to the EV1000™ Clinical Platform monitoring system (Edwards Lifesciences). Thermo-dilution measurements were performed in sets of at least three consecutive injections of 20 mL cold saline (NaCl 0.9%) each, randomly distributed over the respiratory cycle. As required by the EV1000™ software, individual boluses of each set were manually validated by the attending physician before they were included in the data set. By protocol, boluses differing by > 15% of the set average were excluded from the analysis. An EVLWi ≥ 10 mL/Kg was considered as a marker of pulmonary edema and a pulmonary vascular permeability index (PVPI) ≥ 3 diagnostic for ARDS [20].

### Statistical analysis

Data are presented as mean ± standard error, median [IQR], counts and percentages. The Shapiro–Wilk test was used to evaluate normal distributions. The Mann–Whitney U tests were used to compare continuous variables between two groups. The Kruskal–Wallis rank sum test was used to compare continuous variables between three groups in one-way ANOVA models, with the Dunn’s test for post hoc pairwise comparisons. The Receiver Operating Characteristic (ROC) curves were used to show the diagnostic ability of each GLCM feature. The numeric value of area under the ROC curve (AUC) with the trapezoidal rule was calculated for each curve. The AUC values from 0.50 to 0.70 are considered as low accuracy, from 0.70 to 0.90 as moderate accuracy, and > 0.90 as high accuracy. The cut-off points that maximized sensitivity and specificity were calculated in each ROC curve, according to Youden’s J statistic. These parameters coincide with the proportion of true positive (sensitivity) and true negative (specificity) cases that are correctly identified, respectively [23]. A fourfold cross-validation (CV) was performed to evaluate classification error rate in the AUC estimates. The AUCs of two ROC curves were compared by bootstrap test, with 2000 replicates of raw data resampling. Inter-observer variability was tested by intraclass correlation coefficient (ICC) in two-way models for agreement. The Cronbach reliability coefficient was provided as a further measurement of internal consistency. Statistical significance was assumed in each test with *P* value < 0.05. Statistical analyses were carried out using SPSS 20.0 (SPSS, Chicago, IL, USA) and R software/environment (version 3.6.1; R Foundation for Statistical Computing, Vienna, Austria) with the pROC R package. [24].

## Results

We prospectively evaluated 24 patients. Sixteen out of 24 (66%) had CPE (mean age 71 ± 16, 6 male) and eight (33%) fulfilled criteria for ARDS (mean age 55 ± 19, 3 male). Cardiac index, stroke volume, systemic vascular resistance index, global ejection fraction and mean arterial pressure were not significantly different between the two groups. Global end-diastolic and intra-thoracic blood volume index were statistically higher in CPE compared with ARDS patients whereas central venous pressure was higher in ARDS than in CPE (Table 2). Twenty-three healthy subjects (49%) were used as controls (mean age 40 ± 8, 7 male). Twelve chest areas for each subject were examined with LUS, selecting a representative video clip per area, and extracting from them single-frame pictures, with a final yield of 564 single frames for the subsequent analysis.

**Table 2:** Hemodynamic and thermo-dilution parameters from cardiogenic pulmonary edema and acute respiratory distress syndrome patients

Parameter	ARDS (n = 8)	CPE (n = 16)	*p*
CI	2.60 ± 1.1	3.2 ± 0.91	0.165
SVI	34 ± 19	40 ± 14	0.408
SVRI	2442 ± 1161	1859 ± 616	0.223
GEDI	657 ± 230	829 ± 148	0.082
ITBVI	1560 ± 747	2093 ± 547	0.083
EVLWI	16 ± 8.4	15 ± 3.1	0.406
PVPI	3.6 ± 0.34	2.3 ± 0.38	< 0.001
GEF	20 ± 4.1	19 ± 5.8	0.872
MAP	80 ± 18	82 ± 15	0.850
CVP	16 ± 5	11 ± 2	0.001

*ARDS* acute respiratory distress syndrome, *CPE* cardiogenic pulmonary edema, *CI* cardiac index, *SVI* stroke volume index, *SVRI* systemic vascular resistance index, *GEDI* global end diastolic index, *ITBVI* intra-thoracic blood volume index, *EVLWI* extra vascular lung water index, *PVPI* pulmonary vascular permeability index, *GEF* global ejection fraction, *MAP* mean artery pressure, *CVP* central venous pressure

### Comparison between acute respiratory failure patients and healthy control group

There were statistically significant differences between the group with acute respiratory failure (ARDS and CPE) and the healthy control group (HCG) in 7 out of 11 gray-level co-occurrence matrix features: entropy, mean, sum of mean, sum of entropy, and sum of variance were higher in the whole patients’ group than in control group, whereas cluster shade and energy were lower [Electronic Supplementary Material (ESM) Table 1]. There were no differences between groups as concerns contrast, variance, correlation and homogeneity [ESM Table 1, ESM Fig. 1]. By ROC analysis, sum of variance and cluster shade showed the best diagnostic accuracy (AUC = 0.841; *P* < 0.001) with a high statistical power (ESM Table 2, ESM Fig. 2). The classification error rate for AUC evaluated by CV was from 0.095 to 0.097.

### Comparison between acute respiratory failure subgroups and healthy control group

By comparing ARDS and CPE patient subgroups with the HCG, the one-way ANOVA models found a statistical significance in 9 out 11 gray-level co-occurrence matrix features (p < 0.001—ESM Table 3, ESM Fig. 3). The post hoc pairwise comparisons found statistical significance within each matrix feature for ARDS *vs*. CPE and CPE *vs*. HCG, while for ARDS *vs*. HCG a statistical significance occurred only in two matrix features (correlation: *P* = 0.005; homogeneity, *P* = 0.048) (ESM Table 4).

### Comparison between ARDS and CPE subgroups

**Fig. 2:**
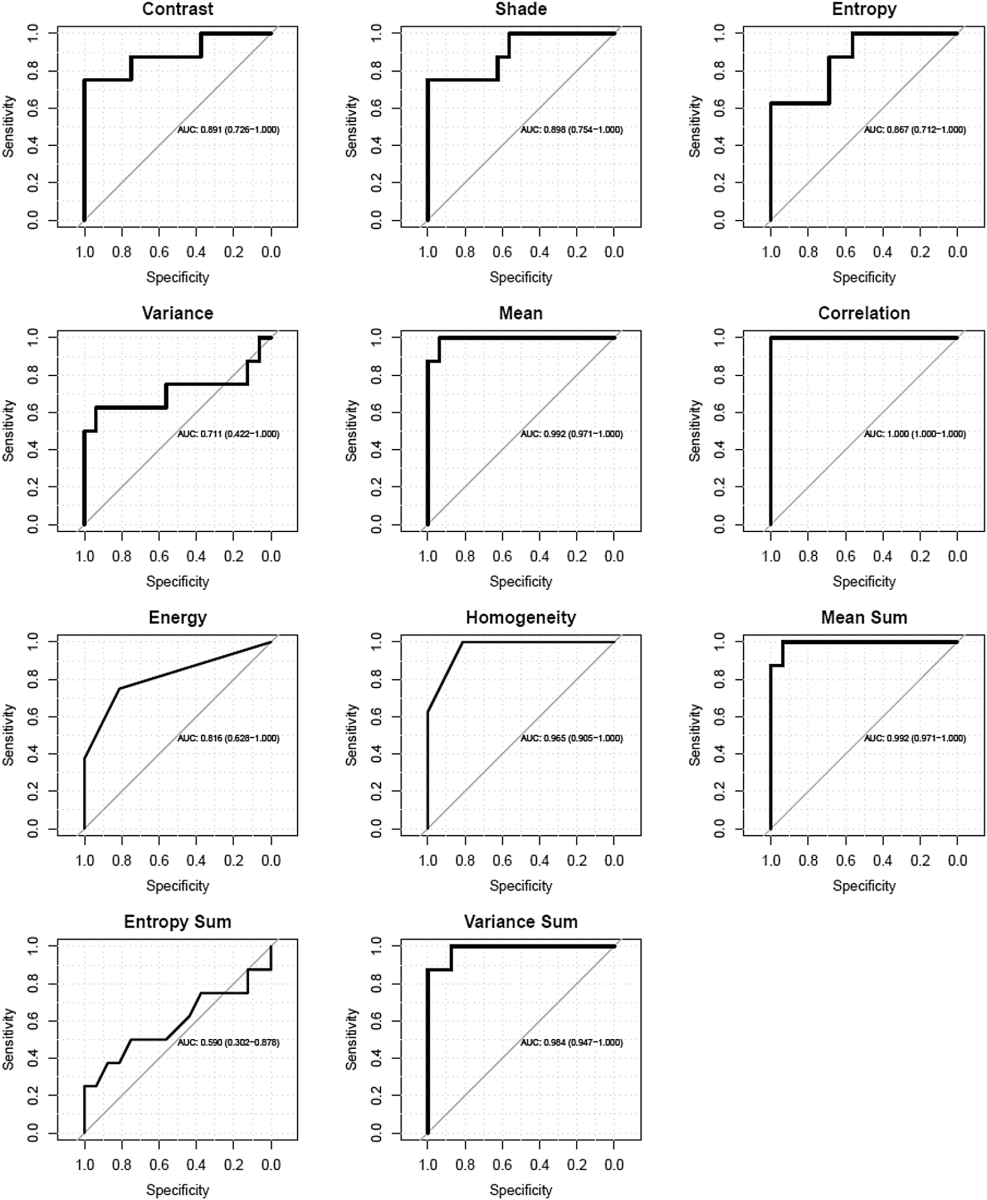
ROC curves of texture features in differentiating acute pulmonary edema and acute respiratory distress syndrome ultrasound patterns

There were statistically significant differences between ARDS and CPE subgroups in 9 out of 11 gray-level co-occurrence matrix features (Table 3). Cluster shade, correlation, energy, and homogeneity were higher in the ARDS than CPE subgroup, whereas contrast, entropy, mean, sum of mean, and sum of variance were lower. There were no statistically significant differences between subgroups for variance and sum of entropy (Table 3, ESM Fig. 4). By ROC analysis, the best diagnostic accuracy occurred for correlation, mean, mean sum and variance sum, with the AUCs ranged from 1.000 to 0.984 (Table 4, Fig. 2). The classification error rate for AUC evaluated by CV was from 0.089 to 0.109.

**Table 3:** Comparison of texture features (mean ± SD) between patients with cardiogenic pulmonary edema and with acute respiratory distress syndrome

GLCM feature	ARDS (n = 8)	CPE (n = 16)	*p*
Contrast	6.27 ± 2.76	10.72 ± 2.26	0.002
Cluster Shade	104.13 ± 114.69	− 56.22 ± 45.58	0.005
Entropy	4.00 ± 0.21	4.26 ± 0.11	0.009
Variance	23.11 ± 6.24	18.32 ± 2.46	0.069
Mean	5.79 ± 1.26	8.87 ± 0.89	< 0.001
Correlation	0.88 ± 0.03	0.74 ± 0.06	< 0.001
Energy	0.03 ± 0.01	0.02 ± 0.01	0.015
Homogeneity	0.65 ± 0.04	0.56 ± 0.03	< 0.001
Mean Sum	11.58 ± 2.53	17.73 ± 1.77	< 0.001
Entropy Sum	3.09 ± 0.13	3.06 ± 0.07	0.590
Variance Sum	125.30 ± 45.16	252.05 ± 52.62	< 0.001

*GLCM Feature* gray level co-occurrence matrices, *CPE* cardiogenic pulmonary edema; *ARDS* acute respiratory distress syndrome

**Table 4:** Diagnostic accuracy of texture features in differentiating acute pulmonary edema and acute respiratory distress syndrome ultrasound patterns

GLCM Feature	AUROC	CI	Cut-off	Sensitivity	Specificity	*p*
Contrast	0.891	0.726–1.000	6.970	1.000	0.750	0.002
Cluster shade	0.898	0.754–1.000	36.46	1.000	0.750	0.002
Entropy	0.867	0.712–1.000	4.085	1.000	0.625	0.004
Variance	0.711	0.422–1.000	21.695	0.938	0.625	0.098
Mean	0.992	0.971–1.000	7.775	0.938	1.000	< 0.001
Correlation	1.000	1.000–1.000	0.810	1.000	1.000	< 0.001
Energy	0.816	0.628–1.000	0.025	0.812	0.750	0.002
Homogeneity	0.965	0.905–1.000	0.590	0.812	1.000	< 0.001
Mean sum	0.992	0.971–1.000	15.515	0.938	1.000	< 0.001
Entropy sum	0.590	0.302–0.878	3.115	0.750	0.500	0.462
Variance sum	0.984	0.947–1.000	163.48	1.000	0.875	< 0.001

*GLCM Feature* gray level co-occurrence matrices, *AUROC* area under receiver operating curve, *CI* confidence intervals, *p* statistical significance of each ROC curve

### Interobserver variability analysis

Inter-observer variability according to intraclass correlation and Cronbach-α reliability coefficient were not clinically significant. Intraclass correlation coefficient for inter-observer variability was 0.951 (95% CI 0.889–0.979; *P* < 0.001), with Cronbach-α reliability coefficient of 0.951.

## Discussion

Our results demonstrated a high diagnostic accuracy of grey-scale texture analysis of LUS images in differentiating patients with severe respiratory failure due to ARDS or hydrostatic pulmonary edema, confirming a more heterogeneous features of pleural lines in the former. This finding can be explained by two mechanisms. The greater derangement of pleural structure associated to inflammatory processes which reflects the correlation between the histological sub-pleural structure and the pleural LUS appearance. Secondly, the different pathophysiology of extravascular lung water distribution in CPE and ARDS edema. ARDS is characterized by an heterogeneous distribution of the disease and thus of the alveolar-capillary membrane leakage leading to a typical inhomogeneous pattern of the pleural line from the very beginning [25, 26]. On the contrary, in CPE, the increased interstitial fluid initially flows proximally from the periphery of the lung to the pulmonary hilum, expanding the lymphatic vessels with a relative preservation of the sub-pleural structure [27]. The analysis of gray-level co-occurrence matrix features allow to add important information to the semiotics based on B-lines, generically identifying the distribution and severity of interstitial syndrome, explaining the relationships between the acoustic signs and the subpleural ultrasonographic features.

Visual assessment of LUS images can be challenging, because ultrasounds can give strong or weak reflections, depending on size and direction of the ultrasound beam, and pleural lines may have an inhomogeneous, speckled appearance both in CPE and in ARDS.

The strength of this approach is that is based on objective grey-scale texture analysis in order to overcome the limitations due to the inter-operator variability [12], the degree of expertise required in analyzing the images and the differences among ultrasound systems hardware, software, and settings [28–30].

The method here described is based on digital pattern recognition, and all texture features were defined based on calculations of close pixel interactions on DICOM format images (Fig. 1). Thus, this approach is completely independent of the specific ultrasound machine post-processing settings that different examiners might use to achieve an adequate ultrasound image. It is also independent of the shape and area of the region of interest selected, because the analysis is not based on morphological characteristics, but on texture features. Second-order grey-scale texture analysis showed a good diagnostic accuracy with the clinical diagnosis, and was able to predict the subsequent diagnosis of ARF in a substantial proportion of cases.

The strength of the study is that all the patients were classified in CPE or ARDS according with the different etiology of the respiratory failure being alternatively cardiogenic or septic shock finally confirmed by the reference gold standard of thermo-dilution technique. All patients had a measured EVLW indexed by predicted body weight > 10 mL/kg expression of a clinically significant pulmonary edema. CPE was characterized by an increase in global end-diastolic index and intra-thoracic blood volume whereas ARDS patients experienced higher values of pulmonary vascular permeability indexes and central venous pressures with a trend towards higher systemic vascular resistances. The remarkable increase of central venous pressure in ARDS patients can be explained by different mechanisms: right ventricular afterload increased (due to both the pathophysiology of ARDS per se and the requirement of positive pressure ventilation); volume replacement and preload centralization (due to vasopressors infusion) related to the application of sepsis bundle guidelines [31].

Some limitations of our study must be pointed out. First, only single frame images were studied, possibly introducing some subjective bias in the frame selection, and in the more limited amount of information in comparison to studying multiple frames. Future technical improvements in the software in order to include real-time multi-frame analysis of pleural lines are currently in the development phase. Secondly, the sample size of our exploratory study is limited low. This limitation influenced the CV approach, where the classification error rate may be under/overestimated due to fourfold CV. We acknowledge the preliminary nature of our work, that does not demonstrate yet the clinical applicability of this new type of ultrasound analysis, but shows very potentially promising results in terms of potential in for discriminating between acute CPE and ARDS.

## Conclusions

The method proposed, based on manual delineation of pleural lines and texture analysis with second-order statistics on LUS images, provides good diagnostic accuracy in differentiating acute CPE and ARDS in ARF patients admitted to the ICU. This image analysis has the potential to support pulmonary edema differential diagnosis, especially when in clinically suspected ARDS LUS images are inconclusive and other diagnostic tools may be unavailable.

## Electronic supplementary material

Below is the link to the electronic supplementary material.

Supplementary material 1 (DOCX 509 kb)

## Data Availability

The dataset analyzed during the current study is available from the corresponding author on reasonable request.

## Abbreviations

ARDS: Acute respiratory distress syndrome
CPE: Cardiogenic pulmonary edema
GLCM: Gray-level co-occurrence matrix
LUS: Lung ultrasound
ICU: Intensive care unit
HCG: Healthy control group
EVLW: Extravascular lung water
LV: Left ventricular
PVPI: Pulmonary vascular permeability index
AUC: Area under the ROC curve
