# Supplementary material for: Second-order grey-scale texture analysis of pleural ultrasound images to differentiate acute respiratory distress syndrome and cardiogenic pulmonary edema
Source: J Clin Monit Comput. 2020 Dec 12;36(1):131–40. doi: 10.1007/s10877-020-00629-1 (PMC8894303; doi:10.1007/s10877-020-00629-1)
Supplement: Supplementary file 1 — Supplementary material 1 (DOCX 509 kb) [file 10877_2020_629_MOESM1_ESM.docx]

**ELECTRONIC SUPPLEMENTARY MATERIAL**

**Second-order grey-scale texture analysis of pleural ultrasound images to differentiate acute respiratory distress syndrome and cardiogenic pulmonary edema**

Claudia Brusasco, Gregorio Santori, Guido Tavazzi, Gabriele Via, Chiara Robba, Luna Gargani, Francesco Mojoli, Silvia Mongodi, Elisa Bruzzo, Rosella Trò, Patrizia Boccacci, Alessandro Isirdi, Francesco Forfori, Francesco Corradi and the UCARE (Ultrasound in Critical care and Anesthesia Research Group).

**Table of Contents**

**ESM Figure 1** Box plots for comparison of each texture feature between acute respiratory failure group (ARFG) and healthy control group (HCG). [p. 2]

**ESM Figure 2** ROC curves of texture features in differentiating healthy controls and acute respiratory failure patients. [p. 3]

**ESM Figure 3** Box plots for comparison of each texture feature between patients with acute respiratory distress syndrome (ARDS), cardiogenic pulmonary edema (CPE) and healthy control group (HCG). [p. 4]

**ESM Figure 4** Box plots for comparison of each texture feature between patients with acute respiratory distress syndrome (ARDS) and cardiogenic pulmonary edema (CPE). [p. 5]

**ESM Table 1** Comparison of texture features (mean ± SD) between healthy controls and acute respiratory failure patients. [p. 6]

**ESM Table 2** Diagnostic accuracy of texture features in differentiating healthy controls and acute respiratory failure patients. [p. 7]

**ESM Table 3** Comparison of texture features (mean ± SD) between acute respiratory failure patient subgroups and healthy control group. [p. 8]

**ESM Table 4** Comparison of texture features between acute respiratory failure patient subgroups and healthy control group: *Post-hoc* test for the significant one-way ANOVA models. [p. 9]

**ESM Figure 1.** Box plots for comparison of each texture feature between acute respiratory failure group (ARFG) and healthy control group (HCG).


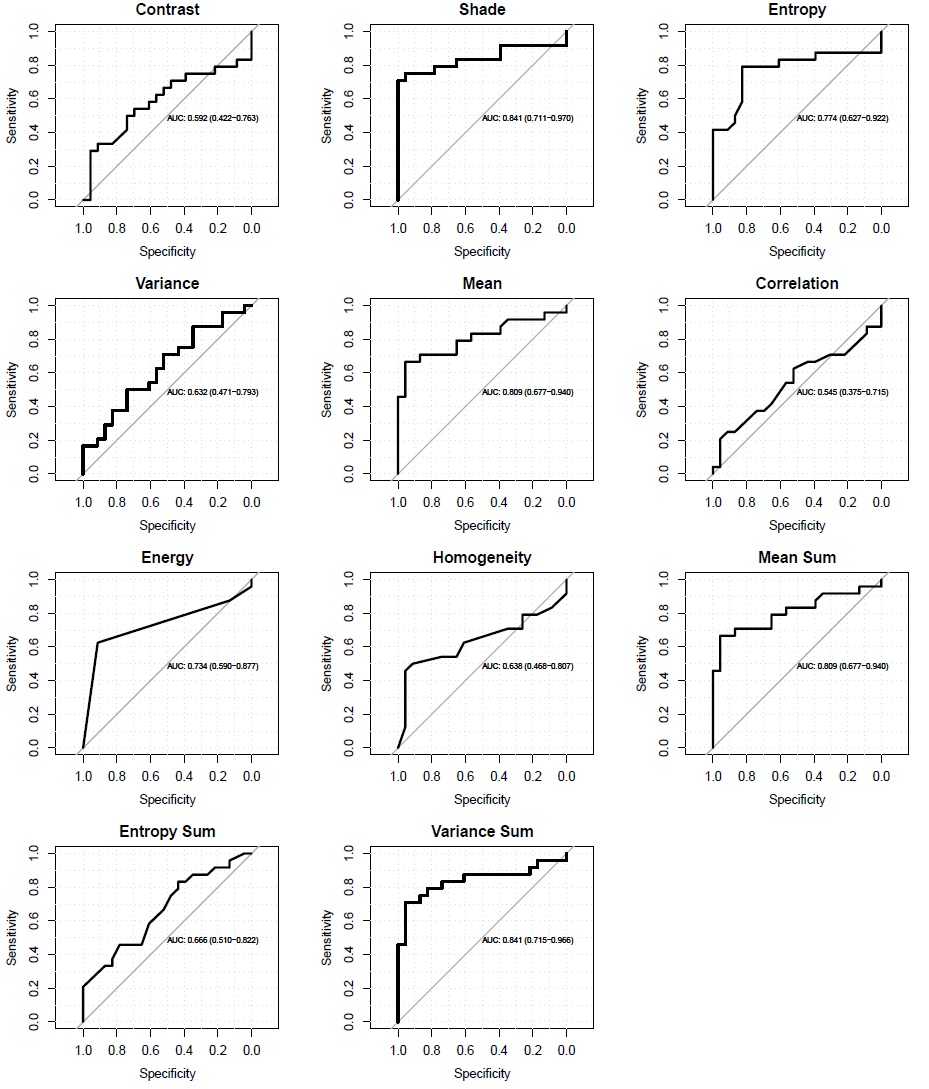


**ESM Figure 2.** ROC curves of texture features in differentiating healthy controls and acute respiratory failure patients**.**

**ESM Figure 3.** Box plots for comparison of each texture feature between patients with acute respiratory distress syndrome (ARDS), cardiogenic pulmonary edema (CPE) and healthy control group (HCG).

**ESM Figure 4.** Box plots for comparison of each texture feature between patients with acute respiratory distress syndrome (ARDS) and cardiogenic pulmonary edema (CPE).

**ESM Table 1**. Comparison of texture features (mean ± SD) between healthy controls and acute respiratory failure patients.

| **GLCM Feature** | **ARFG**  **(n = 24)** | **HCG**  **(n = 23)** | ***p*** |
| --- | --- | --- | --- |
| Contrast | 9.24 ± 3.20 | 8.72 ± 2.60 | 0.544 |
| Cluster Shade | - 2.77 ± 106.40 | 97.41 ± 47.31 | <0.001 |
| Entropy | 4.16 ± 0.19 | 4.05 ± 0.09 | 0.008 |
| Variance | 19.92 ± 4.59 | 17.80 ± 2.77 | 0.062 |
| Mean | 7.84 ± 1.79 | 6.05 ± 1.08 | <0.001 |
| Correlation | 0.78 ± 0.08 | 0.79 ± 0.06 | 0.801 |
| Energy | 0.02 ± 0.01 | 0.03 ± 0.01 | 0.017 |
| Homogeneity | 0.59 ± 0.05 | 0.61 ± 0.03 | 0.165 |
| Mean Sum | 15.68 ± 3.57 | 12.10 ± 2.15 | <0.001 |
| Entropy Sum | 3.07 ± 0.09 | 3.01 ± 0.09 | 0.045 |
| Variance Sum | 209.80 ± 78.43 | 122.75 ± 36.91 | <0.001 |

GLCM Features: gray level co-occurrence matrices; ARFG: acute respiratory failure group; HCG: healthy control group.

**ESM Table 2**. Diagnostic accuracy of texture features in differentiating healthy controls and acute respiratory failure patients.

| **GLCM Feature** | **AUROC** | **CI** | **Cut-off** | **Sensitivity** | **Specificity** | ***p*** |
| --- | --- | --- | --- | --- | --- | --- |
| Contrast | 0.592 | 0.422-0.763 | 11.270 | 0.957 | 0.292 | 0.278 |
| Cluster Shade | 0.841 | 0.711-0.970 | -1.530 | 1.000 | 0.708 | <0.001 |
| Entropy | 0.774 | 0.627-0.922 | 4.105 | 0.826 | 0.792 | 0.001 |
| Variance | 0.632 | 0.471-0.793 | 19.195 | 0.739 | 0.500 | 0.120 |
| Mean | 0.809 | 0.677-0.940 | 7.580 | 0.957 | 0.667 | <0.001 |
| Correlation | 0.545 | 0.375-0.715 | 0.695 | 0.957 | 0.208 | 0.782 |
| Energy | 0.734 | 0.590-0.877 | 0.025 | 0.913 | 0.625 | 0.001 |
| Homogeneity | 0.638 | 0.468-0.807 | 0.565 | 0.957 | 0.458 | 0.097 |
| Mean Sum | 0.809 | 0.677-0.940 | 15.165 | 0.957 | 0.667 | <0.001 |
| Entropy Sum | 0.666 | 0.510-0.822 | 3.015 | 0.435 | 0.833 | 0.050 |
| Variance Sum | 0.841 | 0.715-0.966 | 171.12 | 0.957 | 0.708 | <0.001 |

GLCM Feature: gray level co-occurrence matrices; AUROC: area under receiver operating curve; CI: confidence intervals;

p, statistical significance of each ROC curve.

**ESM Table 3.** Comparison of texture features (mean ± SD) between acute respiratory failure patient subgroups and healthy control group.

| **GLCM Feature** | **ARDS**  **(n = 8)** | **CPE**  **(n = 16)** | **HCG**  **(n = 23)** | ***p*** |
| --- | --- | --- | --- | --- |
| Contrast | 6.27 ± 2.76 | 10.72 ± 2.26 | 8.72 ± 2.60 | <0.001 |
| Cluster Shade | 104.13 ± 114.69 | - 56.22 ± 45.58 | 97.41 ± 47.31 | <0.001 |
| Entropy | 4.00 ± 0.21 | 4.26 ± 0.11 | 4.05 ± 0.09 | <0.001 |
| Variance | 23.11 ± 6.24 | 18.32 ± 2.46 | 17.80 ± 2.77 | 0.092 |
| Mean | 5.79 ± 1.26 | 8.87 ± 0.89 | 6.05 ± 1.08 | <0.001 |
| Correlation | 0.88 ± 0.03 | 0.74 ± 0.06 | 0.79 ± 0.06 | <0.001 |
| Energy | 0.03 ± 0.01 | 0.02 ± 0.01 | 0.03 ± 0.01 | <0.001 |
| Homogeneity | 0.65 ± 0.04 | 0.56 ± 0.03 | 0.61 ± 0.03 | <0.001 |
| Mean Sum | 11.58 ± 2.53 | 17.73 ± 1.77 | 12.10 ± 2.15 | <0.001 |
| Entropy Sum | 3.09 ± 0.13 | 3.06 ± 0.07 | 3.01 ± 0.09 | 0.131 |
| Variance Sum | 125.30 ± 45.16 | 252.05 ± 52.62 | 122.75 ± 36.91 | <0.001 |

GLCM Feature: gray level co-occurrence matrice; ARDS: acute respiratory distress syndrome; CPE: cardiogenic pulmonary edema; HCG: healthy control group.

**ESM Table 4.** Comparison of texture features between acute respiratory failure patient subgroups and healthy control group: *Post-hoc* test for the significant one-way ANOVA models.

| **GMLC Feature** | ***p*** |
| --- | --- |
| Contrast | **ARDS** **CPE**  **CPE** <0.001 ‒  **HCG** 0.071 <0.001 |
| Cluster Shade | **ARDS** **CPE**  **CPE** <0.001 ‒  **HCG** 0.667 <0.001 |
| Entropy | **ARDS** **CPE**  **CPE** <0.001 ‒  **HCG** 0.976 <0.001 |
| Mean | **ARDS** **CPE**  **CPE** <0.001 ‒  **HCG** 0.820 <0.001 |
| Correlation | **ARDS** **CPE**  **CPE** <0.001 ‒  **HCG** 0.005 0.013 |
| Energy | **ARDS** **CPE**  **CPE** 0.001 ‒  **HCG** 0.909 <0.001 |
| Homogeneity | **ARDS** **CPE**  **CPE** <0.001 ‒  **HCG** 0.048 <0.001 |
| Mean Sum | **ARDS** **CPE**  **CPE** <0.001 ‒  **HCG** 0.821 <0.001 |
| Variance Sum | **ARDS** **CPE**  **CPE** <0.001 ‒  **HCG** 0.783 <0.001 |

GMLC Feature: gray level co-occurrence matrices; ARDS: acute respiratory distress syndrome; CPE: cardiogenic pulmonary edema; HCG: healthy control group.
